# The effect of family doctor policy practice on primary health-care workers’ health in Hongkou District of Shanghai, China: varied by occupational divisions?

**DOI:** 10.1186/s12875-020-01275-x

**Published:** 2020-09-30

**Authors:** Jiaoling Huang, Xin Gong, Qing Gu, Rui Liu, Jianwei Shi, Wenya Yu, Ping Zu, Xiaojun Ma, Jie Lin, Jin Sun, Yonghua Yang, Zhaoxin Wang

**Affiliations:** 1 School of Public Health, Shanghai Jiao Tong University School of Medicine, Shanghai, 200025 China; 2 Shanghai Tenth People’s Hospital, Tongji University School of Medicine, Shanghai, 200003 China; 3 Shanghai East Hospital affiliated to Tongji University School of Medicine, Shanghai, 200120 China; 4 Beiwaitan Community Health Service Center of Hongkou Area, Shanghai, 200082 China; 5 Shanghai Municipal Centre for Disease Control and Prevention, Shanghai, 200336 China; 6 General Practice Center, Nanhai Hospital, Southern Medical University, Foshan, 528244 Guangdong China

**Keywords:** Primary care, Occupational health, Family doctor, China

## Abstract

**Background:**

A key component of the 2009 medical reform in China was the change to family doctor (FD) policy practice. However, this led to an increased workload for primary health-care workers (PHCWs) at community health service centres. Their increasing workload may play a significant role in affecting PHCWs’ health.

**Methods:**

A questionnaire survey was conducted in Hongkou district of Shanghai amongst PHCWs including family doctors (FDs), family nurses (FNs), public health doctors (PHDs), and other PHCWs in early 2019. Ordered logistic regression models (Models 1 to 3) were performed to explore the differing health status amongst PHCWs, and their respective influential factors were also tested (Models 4 to 7).

**Results:**

Five hundred sixty-two valid questionnaires were collected with a response rate of 96.4%. Other PHCWs’ (OR = 2.03; 95% CI: 1.163–3.560) and FNs’ (OR = 1.98; 95% CI: 1.136–3.452) self-rated health (SRH) were significantly better than that of FDs. In terms of FNs, the OR of SRH for those who strongly perceived the extra workload brought by FD-contracted services was only 12.0% (95% CI: 0.018–0.815) of that of the no-pressure group. Similarly, FNs with stronger work pressure had worse SRH, i.e., compared with “no” pressure, the SRH ORs for “neutral,” “strong,” and “very strong” evaluations of work pressure were 0.002 (95% CI: 0.000–0.055), 0.001 (95% CI: 0.000–0.033), and 0.000 (95% CI: 0.000–0.006), respectively. Information technology (IT) systems and performance incentives were suggested to improve SRH for FNs, while the former was found to be negatively correlated with other PHCWs. After one unit increase in the PHDs’ team/department support, their OR was 10.7 times (95% CI: 1.700–67.352) higher. In addition, policy support had a negative effect on SRH for PHDs. The OR of “good” assessments of cultural environments was 25.98 times (95% CI: 1.391–485.186) higher than that of “very poor” for Other PHCWs.

**Conclusions:**

The influences of FD policy practice on FNs’ SRH were the most significant amongst PHCWs, rather than FDs’ as expected. The significant factors of SRH were varied over different occupational categories, that is team/department support and policy support (though negative) for PHDs, IT system and incentive for FNs, facility and equipment for FDs, and culture environment for other PHCWs respectively.

## Background

In 2009, a new round of health-care reform was launched in China. A key target of this reform was to improve the population’s poor access to health-care resources by reducing its expensive cost [1]. One of the strategies of this reform was to strengthen the primary health-care system [2, 3]. Hence, primary health care has seen rapid development over the last 10 years [4]. The developmental stages of primary care could be identified, i.e., facility and equipment construction, primary health-care human resource cultivation, contracted services provision, and primary health-care workers’ (PHCWs) team cooperation patterns. In the third stage, several support policies were implemented to achieve the contract rate [5]. China set the ambitious goal that by 2017, the family doctor (FD)-contracted rate should reach 30% for all and 60% for the key population, including elderly, patients with non-communicable disease (NCD), pregnant women, infants, and disabled. In the case of Shanghai, the newly updated number of FD contracts was over 7,000,000 as revealed during the conference of Shanghai Medical Reform Work Promotion in July, 2019 [6]. A study listed 32 detailed FD-contracted services to be supplied by FDs and their assistants in Jiading district of Shanghai, including health evaluations, health management, health record updates, extended prescriptions, long prescriptions for patients with NCD, data collection and reports, family inpatient services, physical health examinations for the elderly, and follow-up management of patients with NCD or the disabled [7]. PHCWs are facing unprecedented numbers of visits and workloads especially in Shanghai, one of the metropolitan cities where the FD policy was first implemented. The Yearbook showed that from 2010 to 2017, the visits per PHCW per day in Shanghai was 28.2, while the averages for western, central, and eastern areas were 10.9, 8.9, and 20.1, respectively [8].

Thus, the fourth stage of the development of community health service centres (CHSCs) in China focused on cooperation amongst PHCWs to cope with the increased number of patients and larger population for health management, especially amongst FDs, family nurses (FNs), and public health doctors (PHDs). It is worth noting that technologists, pharmacists, psychological consultants, dietitians, and rehabilitation therapists working in CHSCs were also included in PHCWs, as the government believed that they played significant role to support or directly provide FD-contracted services. CHSC was the tier-1 hospital in China, through which patients could be referred to specialists in tier-2 and tier-3 hospitals [3]. FDs were mainly responsible for clinical treatment for all patients and health management for the contracted residents, FNs were for clinical nursing and always regarded as FDs’ assistants, and PHDs were for public health issues including vaccination, maternal and child health, infectious diseases and major public health emergencies. However, there are wide variations in the scope of functions and the division of labour amongst PHCWs, and many studies focus on division of responsibilities and cooperation among PHCWs [9–11]. Although different work patterns were practiced across Shanghai or even China, the FD-dominant pattern was outstanding, in which FNs and PHDs were regarded as significant team support. Though we found new team member roles, such as the FD’s neighbourhood assistant whose main work responsibility is to help FDs to connect closely with residents, were emerging, the FD-dominated pattern had not yet been replaced. This finding suggested that FDs might have increased workloads and pressures because of their cooperation with other FD team members.

This increasing workload might play a significant role in affecting PHCWs’ health, but this area has not yet been studied in China. Several studies have focused on work pressures and concluded that PHCWs, especially FDs, were under high work stress [12]. For example, Shen and colleagues found that amongst 308 respondents to a survey conducted in Shanghai, 190 (61.7%) experienced excessive stress, 72 (23.4%) had moderate stress, and 46 (14.9%) had low stress [13]. Little attention was paid to PHCWs’ health, especially from a workload and stress perspective. Xie and colleagues tried to describe the FDs’ workload, work stress, and health status. And negative relationship was found between stress source factor and self-rated health, however, multiple regression model controlling for more variables was not performed [14]. However, the relationship between work stress and physicians’ health has already been discussed widely globally, and scholars have reached a consensus that job stress was a significant factor influencing physicians’ physical and mental health [15]. For example, Buddeberg-Fischer and colleagues found that the groups experiencing constant and increasing extrinsic and intrinsic stress exhibit significantly worse health and life satisfaction compared with the remaining groups after controlling for gender and baseline health [16]. Except for workload and stress, three other predictors were found in these studies, i.e., work support, workplace conditions, and cooperation. A study conducted by Bergman and colleagues found that the physicians’ somatic symptoms were correlated with workload, time spent working, satisfaction, support for stress, and coping abilities. A stepwise regression analysis also showed that excessive workload and support were significant contributors to job stress for men and women [17]. Williams and colleagues conducted a nationwide survey and found that job stress had a powerful influence on physicians’ job satisfaction and physical and mental health. In addition, they found that workplace conditions were also a major determinant of physicians’ well-being [18]. Sarafis et al. also found that occupational stress negatively affected health-related quality of life and conflicts with co-workers was an independent predictive factor [19]. However, workload or stress, workplace conditions or environment, and social or work support have not been considered together in one study.

This study aimed to explore the health status of PHCWs in China and to identify the effect of FDs’ practice on the PHCWs’ health, especially against the background of FD-contracted services since the new round of health-care reform in 2009. We considered occupational categories because we believed that PHCWs’ occupational division might make a difference to their workloads, stress, and health, and workplace conditions, support, workload, and pressure were considered as well as significant factors of PHCWs’ health.

## Materials and methods

### Study design

First, we performed a descriptive health analysis for PHCWs in different occupational categories. A further model analysis was then performed to test whether these occupational categories mattered after controlling for sociodemographic variables. Second, we carefully examined the influential predictors affecting PHCWs’ health, especially the effect of FD practices on PHCWs. The three major categorical groups were tested separately, i.e., FDs, FNs, and PHDs. Based on our literature review, we absorbed the widely accepted factors into our model, including workplace environment/conditions, pressure, cooperation, and support.

### Data collection

A questionnaire survey was conducted in Hongkou District of Shanghai, in which area there are six CHSCs in total. Hongkou district is a very typical central area in Shanghai, which is quite different from other suburban areas in Shanghai. We surveyed all PHCWs in Hongkou District by questionnaire, including FDs, FNs, PHDs, and other PHCWs (technologists, pharmacists, psychological consultants, dietitians, and rehabilitation therapists). It is a population study, but before the survey we also calculated the sample size considering the statistics significance. According to sample size formula of continuous data proposed by Kotrlik JW et al., a suitable sample size should be around 80 (see formula below) [20]. All the significance test had been conducted and gave us strong confidence including Log likelihood, BIC and Pseudo R^2^. We conducted a presurvey interview to revise our questionnaire. A four-part structured questionnaire was then designed to cover sociodemographic information, workload and pressure, cooperation and support, and health status and job satisfaction. In early 2019, our investigators visited each CHSC with help of Community Health Management Centre who was responsible for managing all CHSCs in the Hongkou area.

$$ n=\frac{t^2\ast {s}^2}{d^2}=\frac{1.96^2\ast {0.69}^2}{{\left(5\ast 0.03\right)}^2}=81.29 $$

Where t = value for selected alpha level of 0.025 in each tail = 1.96, s = estimate of standard deviation (SD) in the population = 0.69 (since the population is unknown, we used the sample SD of the five-point scale SRH instead), d = acceptable margin of error for mean being estimated = 0.15. (number of points on primary scale * acceptable margin of error; points on primary scale = 5; acceptable margin of error = .03 [error researcher is willing to except]).

### Variables and measurements

The dependent variable in this study was the PHCWs’ health status. Self-rated health (SRH) was selected as the health variable and defined using a 5-point Likert scale: “How do you evaluate your own health? 1 = very poor, 2 = poor, 3 = neutral, 4 = good, 5 = excellent.” It is widely agreed that this simple global question provides a useful summary of overall health status [21, 22]. FD practices was the key independent predictor that we wanted to examine in this study. Therefore, we divided FD practices into three categories: workload, stress, and support. Workplace environment was the key control variable. Seven items were included in FD practices to cover workload (work time, work intensity, workload in the FD practice), work pressures (work pressure), and cooperation/support (team/department support, CHSCs’ support, and policy support). Support policies refer to preferential policies for family doctors, including investment in facilities and equipment, funding, vocational training, et al. Five items were included in workplace environment measurements: office environment, facilities and equipment, information technology (IT) system, spiritual culture, and performance incentives. We not only included physical workplace environment, but also considered cultural and performance incentives as factors, which were widely discussed as a significant part of the workplace environment [23].

The sociodemographic characteristics included age, gender, marital status, hukou, and occupational category. Education and annual income were also considered as socioeconomic characteristics. Hukou is a special household registration system in China used for population management, especially the migrant population. Hukou is highly related to social welfare because of the huge regional disparity of social wealth in China [24, 25]. (see appendix Table 5).

### Data analysis

Descriptive analyses were performed to capture the sample characteristics, including frequency, percentage, mean, and standard deviation. Health status of occupational categories (FD, FN, PHD and other PHCW) were also described. Ordered logistic regression was performed to examine the SRH differences between different occupation categories, after demographics (Model 1), socioeconomic characteristics (Model 2), and workplace environment (Model 3) were controlled step by step. Based on Model 3, we tested the effect of FD practices on SRH separately for FDs (Model 4), FNs (Model 5), PHDs (Model 6), and Others (Model 7).

## Results

### Sample characteristics

Five hundred ninety-one questionnaires were distributed and 570 returned, with a high response rate of 96.4%, in which 562 questionnaires were valid. The number of FDs, FNs and PHDs were 126 (22.4%), 215 (38.26%), and 72 (12.81%) respectively. The remaining 26.52% in the Others category included technologists, pharmacists, psychological consultants, dietitians, rehabilitation therapists, amongst others, which varied in different CHSCs. Table 1 showed that the average age of this sample was 37.57 years (±8.59 years), and the average age of FD was greater than others. 79.36% of PHCWs were women, while 99.53% of FNs were women. The PHCWs’ martial statuses varied, with 83.10% married, 14.77% single, and only around 2% were divorce or widowed. The majority of PHCWs’ hukou were registered in Shanghai with only 10.50% originally from other provinces. The education levels of the PHCWs was high, with 90.57% having a bachelor’s degree, and 17.46% of FDs had a master’s degree; The average annual income of PHCWs was 8.45 (unit: 10,000 yuan), with FDs (9.19, unit: 10,000 yuan) earning more than other groups.

**Table 1:** Sociodemographic characteristics

Variables	FD		FN		PHD		Other PHW		Total	
	N/ $$ \overline{x} $$	%/± SD	N/ $$ \overline{x} $$	%/± SD	N/ $$ \overline{x} $$	%/± SD	N/ $$ \overline{x} $$	%/± SD	N/ $$ \overline{x} $$	%/± SD
Age	40.03	±8.49	36.37	±7.42	39.87	±8.11	36.10	±9.75	37.57	±8.59
Gender										
Male	49	38.89%	1	0.47%	12	16.67	54	36.24%	116	20.64%
Female	77	61.11%	214	99.53%	60	83.33	95	63.76%	446	79.36%
Marital status										
Single	18	14.29%	18	8.37%	7	9.72%	40	26.85%	83	14.77%
Married	104	82.54%	195	90.70%	63	87.50%	105	70.47%	467	83.10%
Divorced	3	2.38%	1	0.47%	2	2.78%	3	2.01%	9	1.60%
Widowed	1	0.79%	1	0.47%	0	0.00%	1	0.67%	3	0.53%
Hukou										
This subdistrict, Shanghai	30	23.81%	46	21.40%	14	19.44%	11	7.38%	101	17.97%
Other subdistricts, Shanghai	81	64.29%	154	71.63%	54	75.00%	113	75.84%	402	71.53%
Other provinces	15	11.90%	15	6.98%	4	5.56%	25	16.78%	59	10.50%
Education										
High school or below	4	3.17%	16	7.44%	4	5.56%	3	2.01%	27	4.80%
Bachelor degree	100	79.37%	199	92.56%	67	93.06%	143	95.97%	509	90.57%
Master degree or above	22	17.46%	0	0.00%	1	1.39%	3	2.01%	26	4.63%
Annual income, 10,000¥ (Unit: 10,000 $)	9.19 (1.31)	±2.65 (±0.38)	7.98 (1.14)	±1.77 (±0.25)	8.14 (1.16)	±1.82 (±0.26)	8.64 (1.23)	±2.78 (±0.39)	8.45 (1.20)	±2.33 (±0.33)
Total	126		215		72		149		562	

Notes: “Others” refers to other health-care workers except for those in the main four categories in CHSCs, including technologists, pharmacists, psychological consultants, dietitians, and rehabilitation therapists

### Health status of PHCWs in CHSCs

The descriptive analyses showed significant differences amongst PHCWs in their SRH: e.g., 18.25% of FDs assessed their health as good or excellent, while 20.00% of FNs, 26.39% of PHDs, and 29.06% of Others reported good or excellent health. Nevertheless, PHDs had the worst assessment (poor or very poor) of their own health, which indicated an apparent polarization in their SRH. The more consistent finding was that most of the PHCWs of different occupational categories rated their health as ‘neural’. In general, Other PHCWs and FNs seemed to have better SRH than FDs and PHDs (see Table 2).

**Table 2:** PHCWs’ health status

Self-rated health	Family doctor	Family nurse	Public health doctor	Others
Very poor	0.79%	0.93%	6.94%	0.68%
Poor	15.08%	7.91%	13.89%	10.14%
Neutral	65.87%	71.16%	52.78%	60.14%
Good	15.87%	18.14%	20.83%	25.68%
Excellent	2.38%	1.86%	5.56%	3.38%
Total	126	215	72	148
Chi-squared test	29.3467 (*p* = 0.000)			

We further performed model tests to distinguish health differences amongst PHCWs, controlling for socioeconomic characteristics and workplace environment. We observed an obviously positive relationship between the workplace environment and SRH. Specifically, the SRH of those who evaluated the IT system in their workplace as being “good” were 2.95 times than that of those who reported it as being “very poor.” The SRH of “very good” evaluators of their cultural environment were 4.74 times higher than that of those who reported it as “very poor.” PHCWs who had better evaluations of their performance incentives seemed to have better health, although this relationship did not seem to show a gradient. We then focused on the effect of occupational categories on SRH, which was quite consistent with our descriptive findings. Compared with FDs, Other PHCWs and FNs had better SRH, e.g., the odds ratio (ORs) were 2.03 for Others, 1.98 for FNs, while FDs were the reference group. Besides, we found that males reported better (OR = 2.43, 95%CI: 1.480–3.983) health status than females, and the SRH of PHCWs from other provinces was 2.37 times than that of those local district PHCWs (Table 3).

**Table 3:** Ordered logistic regression of SRH for all PHCWs

Variable	Model 1			Model 2			Model 3		
	OR	*P* value	95% CI	OR	*P* value	95% CI	OR	*P* value	95% CI
**Sociodemographic characteristics**									
**Age**	0.969**	0.007	0.947–0.991	0.98	0.112	0.957**–**1.005	0.985	0.231	0.960**–**1.009
**Gender**	1.886**	0.004	1.222–2.909	2.047**	2.047	1.268**–**3.302	2.428***	0.000	1.480**–**3.983
**Hukou (Ref. = This subdistrict, Shanghai)**									
Other subdistricts, Shanghai	1.429	0.129	0.901–2.263	1.319	0.246	0.826**–**2.105	1.313	0.294	0.789**–**2.184
Other provinces	2.602**	0.006	1.318–5.139	2.612**	0.006	1.312**–**5.200	2.370*	0.022	1.133**–**4.958
**Education (Ref. = High school or below)**									
Bachelor degree				2.029	0.111	0.850**–**4.843	1.866	0.176	0.755**–**4.611
Master degree				3.089	0.076	0.888**–**10.741	3.407	0.065	0.928**–**12.495
**Annual income**				0.977**	0.003	0.962**–**0.992	0.983*	0.032	0.967**–**0.998
**Occupational categories (Ref. = Family doctor)**									
Family nurse				1.950*	0.013	1.154–3.293	1.980*	0.016	1.136–3.452
Public health doctor				1.641	0.140	0.849–3.170	1.593	0.192	0.791–3.210
Others				1.674	0.054	0.992–2.825	2.034*	0.013	1.163–3.560
**Workplace environments**									
**Information technology system (Ref. = very poor)**									
Poor							0.591	0.340	0.199–1.743
Neutral							2.219	0.136	0.777–6.336
Good							2.950*	0.049	1.002–8.675
Very good							2.059	0.194	0.692–6.120
**Cultural environment (Ref. = very poor)**									
Poor							3.519	0.080	0.861–
Neutral							1.711	0.408	0.479–6.106
Good							3.324	0.066	0.925–11.944
Very good							4.738*	0.016	1.329–16.884
**Performance incentive (Ref. = very poor)**									
Poor							2.223	0.061	0.964–5.119
Neutral							2.602*	0.012	1.236–5.476
Good							2.360*	0.044	1.022–5.447
Very good							1.491	0.551	0.401–5.534
Statistics									
N	561			561			558		
Log likelihood	− 552.7			− 544.6			− 508		
BIC	1175			1196.7			1250.1		
Pseudo R^2^	0.0269			0.0411			0.1015		

Notes: * *p* < 0.05; ** *p* < 0.01; *** *p* < 0.001; Insignificant variables including marital status, office environment, and facilities and equipment are hidden to ensure that the table is more simplified and readable

### Factors associated with different PHCWs’ health

We then separated our model into four sub-models focusing on four categories of PHCWs, in which all sociodemographic, socioeconomic, and workplace environment variables were controlled. Some interesting findings were obtained. Workplace environment and FD practices were significant predictors of SRH, although this varied by occupational category. For FDs, the facilities and equipment in the workplace environment were the only significant variable, i.e., the OR for “good” evaluations was 235.73 times than that for “very poor” evaluations. Surprisingly, all items in FD practices did not have a significant impact on the SRH of FDs. Office environment had a negative effect on FNs’ SRH, while IT system and performance incentives positively affected their SRH. A significant effect was observed in FNs from FD practices; specifically, FNs who strongly felt under pressure from their increased workload because of FD-contracted services had worse SRH. That is, the OR for “strong” pressure was 12.0% higher than that of “no” pressure. Similarly, those feeling stronger work pressure had worse SRH, i.e., compared with “no” pressure, the SRH ORs for “neutral,” “strong,” and “very strong” evaluator of work pressure was 0.002, 0.001, and 0.000, respectively. PHDs did not evaluate their workplace environment that significantly, but supported FD practices. Specifically, considering a unit increase in team/department support, the OR was 10.7 times than that from before the increase. However, policy support had a negative effect on the SRH of PHD. Considering Other PHCWs, FD practices did not play significant role in affecting their SRH, but the workplace environment did, especially the cultural environment, facility, and equipment. Specifically, compared with those “very poor” evaluators of facility and equipment, the OR of those who evaluated it as being “poor” was 138.34 times higher. Similarly, those who assessed their cultural environment as being good were better in SRH than those as poor, i.e., the OR for “good” was 25.98 times higher than that of “very poor.” However, a negative relationship was found between IT system and SRH (Table 4).

**Table 4:** Separate ordered logistic regressions of health for different PHCWs

Variable	Model 4 (Family doctor)			Model 5 (Family nurse)			Model 6 (Public health doctor)			Model 7 (Others)		
	OR	*P* value	CI	R	*P* value	95% CI	OR	*P* value	95% CI	OR	*P* value	95% CI
**Sociodemographic characteristics**												
**Age**	0.942	0.173	0.865–1.026	1.018	0.587	0.954–1.086	0.895	0.103	0.783–1.023	1.016	0.577	0.962–1.073
**Gender**	3.048	0.061	0.951–9.770	83.929	0.060	0.824–8545.770	0.113	0.087	0.009–1.376	5.646**	0.001	1.994–15.990
**Education (Ref. = High school or below)**												
Bachelor’s degree	1.918	0.677	0.090–40.963	14.098**	0.004	2.362–84.143	0.334	0.562	0.008–13.591	0.464	0.628	0.021–10.306
Master’s degree	4.388	0.366	0.178–108.383	–	–	–	4.10E+ 05	1	–	0.197	0.485	0.002–18.857
**Annual income**	0.972	0.200	0.931–1.015	0.983	0.378	0.946–1.021	0.858**	0.003	0.776–0.948	1.006	0.764	0.970–1.043
**Workplace environments**												
**Office environment (Ref. = Very poor)**												
Poor	0.218	0.46	0.004–12.405	0.001*	0.003	0.000–0.096	0.207	0.714	0.000–943.872	1.672	0.734	0.087–32.314
Neutral	0.165	0.331	0.004–6.233	0.012	0.053	0.000–1.062	0.333	0.812	0.000–2927.996	0.915	0.938	0.096–8.728
Good	0.05	0.1	0.001–1.769	0.018	0.093	0.000–1.954	0.033	0.401	0.000–95.050	1.077	0.951	0.102–11.394
Very good	0.121	0.288	0.002–5.933	0.014	0.069	0.000–1.398	0.357	0.759	0.000–261.548	1.448	0.778	0.111–18.952
**Facility and equipment (Ref. = very poor)**												
Poor	86.194	0.067	0.729–10,192.560	19.489	0.228	0.156–2439.597	9.80E+ 08	0.998	–	138.335*	0.03	1.601–11,956.5
Neutral	98.905	0.074	0.637–15,346.800	11.415	0.346	0.072–1804.725	2.30E+ 10	0.998	–	4.919	0.395	0.125–193.59
Good	235.733*	0.044	1.158–47,999.460	8.316	0.422	0.047–1458.228	3.40E+ 11	0.997	–	12.233	0.173	0.333–449.672
Very good	89.389	0.101	0.415–19,245.420	7.419	0.424	0.054–1013.823	3.30E+ 09	0.998	–	11.6	0.17	0.350–384.606
**Information technology system (Ref. = very poor)**												
Poor	1.191	0.908	0.061–23.227	21.061*	0.036	1.214–365.342	6.497	0.388	0.093–455.615	0.006***	0.001	0.000–0.119
Neutral	2.273	0.581	0.123–42.074	37.716**	0.008	2.550–557.840	9.427	0.256	0.196–453.069	0.047*	0.028	0.003–0.724
Good	8.584	0.191	0.343–214.654	20.968*	0.027	1.408–312.282	0.544	0.779	0.008–37.761	0.091	0.074	0.007–1.257
Very good	5.573	0.322	0.186–66.734	10.132	0.081	0.752–136.434	13.795	0.188	0.278–683.983	0.051*	0.042	0.003–0.897
**Cultural Environment (Ref. = very poor)**												
Poor	0.004	0.218	0.000–26.694	6.079	0.351	0.137–269.703	23.026	0.194	0.202–2623.828	7.911	0.251	0.232–269.645
Neutral	0.007	0.26	0.000–39.301	0.6	0.799	0.012–30.763	28.227	0.102	0.517–1541.532	1.414	0.81	0.085–23.629
Good	0.003	0.226	0.000–33.659	1.618	0.816	0.028–92.432	2.525	0.458	0.218–29.220	25.980*	0.029	1.391–485.186
Very good	0.01	0.334	0.000–114.362	4.08	0.513	0.061–275.038	–	–	–	13.162	0.098	0.624–277.621
**Performance incentive (Ref. = very poor)**												
Poor	1.933	0.583	0.184–20.310	10.106*	0.026	1.327–76.956	0.148	0.452	0.001–21.335	1.302	0.817	0.139–12.220
Neutral	1.365	0.786	0.144–12.940	10.752*	0.017	1.524–75.866	0.686	0.86	0.011–44.618	1.013	0.989	0.163–6.311
Good	0.911	0.942	0.075–11.052	19.065**	0.007	2.252–161.370	3.923	0.549	0.045–343.590	0.633	0.655	0.085–4.722
Very good	–			0.257	0.356	0.014–4.595	0.048	0.305	0.000–15.836	1.771	0.673	0.124–25.207
**Family Doctor Practice**												
**Workload brought by FD practice (Ref. = none)**												
Tiny	–			0.181	0.115	0.022–1.514	0.069	0.257	0.001–7.005	1.264	0.748	0.302–5.295
Neutral	42.355	0.234	0.089–20,182.05	0.169	0.052	0.028–1.016	0.303	0.556	0.006–16.075	0.848	0.825	0.197–3.657
Strong	13.309	0.437	0.020–9066.505	0.120*	0.030	0.018–0.815	0.151	0.358	0.003–8.497	0.595	0.523	0.120–2.936
Very strong	67.059	0.215	0.087–51,873.88	0.111	0.051	0.012–1.012	0.010	0.086	0.000–1.931	0.179	0.106	0.022–1.441
**Work pressure (Ref. = none)**												
A little	(base)			0.096	0.190	0.003–3.193	0.534	0.799	0.004–67.792	0.596	0.752	0.024–14.782
Neutral	0.409	0.481	0.034–4.902	0.002***	0.000	0.000–0.055	0.611	0.764	0.025–15.160	0.355	0.51	0.016–7.745
Strong	0.157	0.203	0.009–2.725	0.001***	0.000	0.000–0.033	6.049	0.308	0.190–192.356	0.239	0.393	0.009–6.402
Very strong	0.225	0.438	0.005–9.769	0.000***	0.000	0.000–0.006	–	–	–	0.061	0.155	0.001–2.885
**Team/department support**	0.778	0.644	0.269–2.255	0.451	0.109	0.170–1.194	10.700*	0.012	1.700–67.352	0.856	0.741	0.340–2.153
**Policy support**	1.515	0.259	0.736–3.118	0.832	0.718	0.307–2.253	0.155**	0.008	0.039–0.614	1.07	0.883	0.438–2.614
Statistics												
N	124			214			72			148		
Log likelihood	−88.8			− 122.6			−54.4			−102.9		
BIC	413.9			513.6			309.7			460.6		
Pseudo R^2^	0.2668			0.3432			0.4121			0.3284		

Notes: * *p* < 0.05; ** *p* < 0.01; *** *p* < 0.001. Insignificant variables including marital status, hukou, work time, work intensity, CHSC support are hidden to ensure that the table is more simplified and readable

## Discussion

The descriptive analyses and model tests showed significant differences in SRH status amongst the PHCWs. FNs and Other PHCWs had much better SRH than FDs, who played the key role in of FD teams. Studies have not focused on PHCWs’ health to date, although the Chinese government has repeatedly strengthened the significant role of PHCWs to support them using policy [26]. In addition, the health differences amongst FDs, FNs, PHDs, and Other PHCWs have not yet been explored. Worse self-reported health could be related to teamwork in primary health care, where FDs were usually in charge and bore primary responsibility. The relationships between job characteristics, role stress, and health had been documented widely in the psychological literature. For example, Kelloway and Barling proposed and evaluated a causal model that delineated the processes whereby individual mental health was affected by job characteristics and found that indices of job-related affective well-being and subjective competence mediated the relationships between job characteristics and role stressors on the one hand and mental health on the other hand [27].

The health differences amongst PHCWs were explored further and apparent differences were also found. Surprisingly, the significant effect of FD practice was only discovered in FNs, rather than FDs, PHDs, or Others, which was contrary to previous cognition. Specifically, negative relationships with SRH were found in the effect of FD-contracted services workload and work pressure for FNs. In most studies, FDs are described as the dominant leaders of FD-contracted service teams, including PHDs, FNs, and Other team members [28]. FDs were thought to be the greatest contributor to primary health care by the public and social media. However, in our study, FNs were significantly affected by the growing workload in delivering FD-contracted services, in addition to the pressure to work because of their increased workload. FNs usually work closely with FDs as their assistants, although they can work in different ways [29]. As FD-contracted services were implemented, the FNs’ workload grew dramatically in two main components: the usual health care for visiting patients and health management for FD-contracted residents [30]. Pan and Yang analysed different models of FD teams in China. They found that FNs always supported FDs in areas of medical care, health consultation, health examinations, health profile updates, supported PHDs with NCD management, health education, and so on [31]. Using practice evidence from four CHSCs in Shanghai where FNs were regarded as assistants, Lu and colleagues explored the FNs’ work cooperation more deeply [7]. They found there were 32 specific work tasks related to FNs’ daily work, which were classified into four categories: primary medical care, primary public health care, major public health care, and FD-contracted services. This strongly shows that FNs have been deeply involved in primary health care since the 2009 health-care reform, but have also been ignored. Our study drew the preliminary inference that FD-contracted services had led to heavy work pressure for FNs that even caused serious deterioration of their health.

Our study also suggested that an improved IT system and performance incentives could help improve FNs’ SRH. FNs in CHSCs were mainly responsible for medical care, but also regarded as FDs’ assistants in FD contracted services provision. For example, FNs collected each contracted resident’s health information to establish electronic health record, and update them annually, and FNs were also responsible for following-up NCD patients’ health status, all of which were highly dependent on information systems. The Chinese government increased its subsidies to primary health-care institutions from ¥19 billion (US$2.8 billion) in 2008 to ¥140 billion ($20.3 billion) in 2015, as part of China’s new health-care reform initiated in 2009 [32]. Infrastructure, including IT systems, had been improved greatly in the CHSCs because the government developed and deployed a series of IT systems that covered all CHSCs, including systems for infectious diseases and public health emergencies reports, health management for psychosis, resident health records, and electronic medical record systems [2]. A well-established system helps to reduce the difficulty of work and increase work efficiency, thus improving health. A recent study showed that after the application of IT in China, medical costs were obviously reduced, consultation became more convenient, and the health system worked more efficiently [33], which is consistent with previous studies abroad [34, 35]. In our study, self-rated IT system satisfaction was positively correlated with higher SRH for FNs, suggesting a positive effect of IT systems on FNs’ health. And performance incentive was also found to be advantageous for FNs’ health, which was consistent with health disparity theory, revealing that better income, education, and occupation position were positively correlated with health status, which was articulated by Margaret Whitehead [36]. However, the performance incentive and income of FNs in China was criticized by many scholars. A national survey conducted by Li and colleagues showed that the median annual income for doctors who had 2 to 10 years of clinical practice experience after graduating was ¥48,000, ranging from ¥35,000 in the central regions to ¥60,000 in eastern regions, which were lower than the average income in China (¥62,029) [2]. We suggested further improvements to the IT system, especially a further combination of different systems, and construction of performance incentives, especially for income.

In terms of significant factors for FDs, the only significant predictor was facility and equipment, which was also positively related with SRH for Other PHCWs. The relationship between workplace environment and health for doctors has been widely documented and similar results show that a better environment equals better health [37, 38]. Another interesting finding for other PHWCs was that SRH was negatively correlated with IT system rating, which is contrary to our previous finding. “Others” are mainly made up of technologists, pharmacists, psychological consultants, dietitians, and rehabilitation therapists in CHSCs, and a large part of their work is done through web-based systems. For example, medical imaging medical technicians use a computer system to produce test reports. With the upgrading of information system, they may therefore take on more work. Another example is that the daily job for psychologists in CHSCs was online counselling, which is preferred by most of residents, as they believed that it is shameful to have a mental illness, which is obviously stigmatized in China. Therefore, the improvement of the information system has brought more workload to other PHCWs, and therefore their health deteriorates.

First, team/department support played a positive role while policy support played a negative role in PHDs’ SRH. Some studies indicated that PHDs had joined FD teams, but had not assimilated into the team. Most PHDs still maintained their original work patterns, i.e., reporting data to public health institutions. Public health was broadly defined as all social efforts to prevent diseases and improve the health of the population; however, this was usually understood from the perspective of the services or activities provided by public health institutions in China, which differ from medical services [39, 40]. PHDs were affiliated with CHSCs, but reported their work directly to public health institutions, which suggests the fragmented delivery of public health services, and limited cooperation mechanisms in providing integrated care for prevention, treatment, and health promotion [41]. The absence of this cooperation affected PHDs’ SRH, according to our study. Recent studies have provided similar results, showing that the lack of organizational support might result in larger work pressures, a poor sense of integration, and worse health for employees [42]. Besides, the Chinese government and public media had given strong attention to FDs instead of PHDs; therefore, the current policies were not advantageous or supportive for PHDs, which resulted in a weak sense of support from social policies [43].

Second, the cultural environment was a protective factor for Other PHCWs’ health. Other PHCWs were regarded as auxiliary staff for FD-contracted services, but who also performed indispensable work in the daily operation of CHSCs. According to previous studies, building a cultural environment, including team building seminars, regular vocational studies, employee salons, etc., reflected the importance that managers placed on their employees, which could improve employees’ sense of integration and health, also worked for Other PHCWs [44, 45].

### Strength and limitation

There are some limitations still: firstly, our study was a cross-sectional one, through which we could not observe the change of workload and pressure before and after the reform; secondly, this study initially explored whether FD practice might influence PHCWs’ health, but the influential path and mechanism could be explored using method like system dynamics modelling, which could help us to better understand how FD practice affects PHCWs’ health. Thirdly, a larger sample could be better for sub-group analysis, though all the model fit quite well in our study. Finally, Hongkou is a central area in Shanghai, and Shanghai is quite a large city, suggesting that there may be large differences between the regions. Thus, a follow-up and cross-area study is worth conducting, in which the work path and mechanism could be further studies.

## Conclusions

We found that this influence of FD practice was most significant on FN’s health, rather than FDs as expected. We also found that team/department support to PHDs played a positive role, while policy support played a negative role in PHDs’ SRH, suggesting that cooperating well in FD team construction and more supportive policy were needed by PHDs. The significant factor affecting the health of FD and Other PHCWs was the workplace environment, e.g., the facilities/equipment and cultural environment, respectively, which suggests that a better physical and cultural environment is needed.

## Data Availability

The datasets generated and analysed during the present study are not publicly available because of the need to protect privacy. However, they are available from the corresponding author on reasonable request (hkyangyonghua@sina.com).

## Appendix

**Table 5:** Variables and measurements

Variable category	Dimensions	Sub-dimensions	Variables	Item	Measurement
Dependent variable	Health status	–	Self-rated health	How do you evaluate your own health?	1 = Very poor, 2 = Poor, 3 = Neutral, 4 = Good, 5 = Excellent
Key independent predictor	FD practice	Workload	work time	How about your work time?	1 = Never overtime, 2 = Not often overtime,3 = Sometimes overtime, 4 = Often overtime, 5 = Usually overtime
			work intensity	How about your work intensity?	1 = Very weak, 2 = Weak, 3 = Neutral, 4 = Strong, 5 = Very strong
			workload in the FD practice	How about the workload brought by FD practice?	1 = None, 2 = Tiny, 3 = Neutral Strong, 4 = Very strong
		Work pressure	work pressure	How about the work pressure?	1 = None, 2 = A little, 3 = Neutral Strong, 4 = Very strong
		Cooperation and support	team/department support	Please score the team/department support?	[1–5] satisfaction 1 = very poor, 2 = poor, 3 = neutral, 4 = good, 5 = very good
			CHSCs’ support	Please score the CHSC support	[1–5] satisfaction 1 = very poor, 2 = poor, 3 = neutral, 4 = good, 5 = very good
			policy support	Please score the policy support	[1–5] satisfaction 1 = very poor, 2 = poor, 3 = neutral, 4 = good, 5 = very good
Key control variable	Workplace environments	–	Office environment	How about your office environment	1 = Very poor, 2 = Poor, 3 = Neutral, 4 = Good, 5 = Very good
		–	Facilities and equipment	How about your facilities and equipment	1 = Very poor, 2 = Poor, 3 = Neutral, 4 = Good, 5 = Very good
		–	Information technology system	How about your information technology (IT) system	1 = Very poor, 2 = Poor, 3 = Neutral, 4 = Good, 5 = Very good
		–	Cultural environment	How about your cultural environment	1 = Very poor, 2 = Poor, 3 = Neutral, 4 = Good, 5 = Very good
		–	Performance incentive	How about your performance incentive	1 = Very poor, 2 = Poor, 3 = Neutral, 4 = Good, 5 = Very good
	Socio-demographic characteristics	–	Age	When were you born?	[22–67], Unit: years
		–	Gender	What’s your gender?	1 = Male, 0 = Female
		–	Marital status	What is your marital status?	1 = Single, 2 = Married, 3 = Divorced, 4 = Widowed
		–	Education	What is your education level?	1 = High school or below, 2 = Bachelor degree, 3 = Master degree or above
		–	Hukou	What is your household registration?	1 = This subdistrict, Shanghai, 2 = Other subdistricts, Shanghai, 3 = Other provinces
		–	Annual income	What is your annual income?	[1–36], Unit: 10,000 Yuan
		–	Occupational category	What is your occupational category?	1 = FD, 2 = FN,3 = PHD,4 = Others

## Abbreviations

FDs: Family doctors
FNs: Family nurses
PHDs: Public health doctors
CHSCs: Community health service centres
PHCWs: Primary health-care workers
NCD: Noncommunicable diseases
SRH: Self-rated health
OR: Odds ratio
IT: Information technology
